# Taking the cat-and-mouse game to the next level: different perspectives on the introduction of the German New Psychoactive Substances Act

**DOI:** 10.1186/s12954-022-00704-7

**Published:** 2022-11-03

**Authors:** Regina Kühnl, Darya Aydin, Sabine Horn, Sally Olderbak, Uwe Verthein, Ludwig Kraus

**Affiliations:** 1 IFT Institut für Therapieforschung, Leopoldstraße 175, 80804 Munich, Germany; 2 Institute of Psychology and Education, Ulm University, Albert-Einstein-Allee 47, 89069 Ulm, Germany; 3 Unit III.5 Criminal Law, Criminal Procedural Law and Criminal Politics, German Police University, Zum Roten Berge 18–24, 48165 Muenster, Germany; 4 Psychology Department, University of Arizona, 1503 E University Blvd., Tucson, AZ 85721 USA; 5 Department of Psychiatry and Psychotherapy, Centre of Interdisciplinary Addiction Research, University Medical Centre Hamburg-Eppendorf, Martinistraße 52, 20246 Hamburg, Germany; 6 Department of Public Health Sciences, Centre for Social Research on Alcohol and Drugs, Stockholm University, 106 91 Stockholm, Sweden; 7 Institute of Psychology, ELTE Eötvös Loránd University, Egyetem tér 1–3, 1053 Budapest, Hungary

**Keywords:** Drug policy, New psychoactive substances (NPS), New Psychoactive Substances Act (NpSG), Hazardous drug use, Law enforcement, Qualitative research

## Abstract

**Background:**

To counteract the spread of new psychoactive substances (NPS) and to prevent the emergence of novel substances, specifically designed as a response to the legal control of individual substances, a new law was introduced in Germany in 2016: the New Psychoactive Substances Act (NpSG). The NpSG combines a generic approach with the waiver of criminal liability for the acquisition and possession of NPS for personal use, which is a novelty in German narcotics law. The present study aimed at exploring the impact of the introduction of the NpSG from three different perspectives—NPS users, staff of addiction care facilities, and members of law enforcement authorities—to better understand the dynamics surrounding such a change in legislation and to contribute to the body of international experience in dealing with NPS.

**Methods:**

Semi-structured narrative interviews were conducted with a total of 193 representatives of the three different groups affected by the law. These interviews included questions on perceived changes associated with the introduction of the NpSG as well as questions on opinions regarding legal and criminal policy issues. The analysis was carried out using qualitative content analysis according to Mayring.

**Results:**

Most interviewees welcomed the non-criminalisation approach of the NpSG but also noticed that, in practice, not much has changed for users. Nevertheless, the changes in legislation have had an impact on the market. For example, novel substances have emerged circumventing the new legislation. According to users, this has led some to reduce NPS use and others to adopt more hazardous consumption patterns. Overall, most respondents did not expect the introduction of the NpSG to bring any significant changes.

**Conclusions:**

Although the idea of non-criminalisation inherent to the NpSG is appreciated and the generic approach has been well implemented in the law, thus covering a wide range of substances, the introduction of the law—perhaps for that very reason—has also had unintended and negative consequences, taking the cat-and-mouse game to the next level. To end the game, or at least to defuse the game situation, a combination of different strategies will be necessary, with legislation always playing a key role.

## Background

New psychoactive substances (NPS) pose specific challenges to addiction care, law enforcement, and policy-making. The corresponding market comprises an immense spectrum of substances in diverse forms with sometimes very different ranges of psychoactive effects and risk profiles [1]. Since the mid-2000s, the number of different NPS on the market has been steadily increasing, albeit at a slower pace in recent years [2, 3]. By the end of 2021, 1124 different NPS were reported via early warning systems worldwide, of which around 880 were identified in Europe and more than 460 in Germany [3, 4].

As the diversity of available substances and products has increased, so has the diversity of users. These include recreational users who use NPS as (allegedly) legal and easily available cannabis substitutes or as party drugs, psychonauts, and people who intend to self-optimise or self-medicate using NPS [5–9]. Another group of users consists of people who—often because of their criminal past or in an addiction care context—are subjected to regular drug testing and use NPS due to their poor detectability [7]. NPS users also increasingly include people engaged in highly problematic drug use, often experiencing homelessness, for whom the high potency and relatively low price of these substances are usually the decisive factors for use [5–7, 10, 11]. On top of this, a growing number of people use NPS unintentionally. This phenomenon has recently been observed mainly in connection with low-THC cannabis products (in herbal, resin, or e-liquid form) adulterated with synthetic cannabinoids, such as MDMB-4en-PINACA, yet hardly distinguishable from genuine cannabis products in terms of sensory perception [12]. As the synthetic cannabinoids involved are often highly potent substances, these products carry a high risk of poisoning, especially considering that users are most likely unaware of the hazardous ingredients [12, 13].

NPS were initially designed as legal alternatives to substances listed under the UN Drug Conventions of 1961 and 1971 in an attempt to mimic the effects of the original drugs. Some manufacturers are still striving to circumvent the current legislation by specifically adjusting the chemical structure of the substances, usually with only minor modifications, thus introducing numerous new, sometimes actually legal, substances onto the market [13]. Moreover, globalisation and innovative information technologies serve as catalysts for the diffusion of these substances, whose effects and health risks are particularly difficult to assess in absence of sufficient research and contribute to this highly dynamic market [14]. The fact that legal updates can be very time-consuming further complicates responding to newly emerged NPS. In addition, many substances are difficult to detect in laboratory chemical analysis because of a lack of technical or financial resources [15].

To meet these challenges, governments worldwide have adopted different strategies. King (2013) distinguishes between three types of traditional control mechanisms: (1) specific listing, (2) generic definitions, and (3) the analogue approach [16].

*Specific listing*, or the individual listing of substances according to their chemical names, has the advantage of being accompanied by a high degree of legal certainty in criminal law. The major disadvantage is the legislative process for the inclusion of novel substances can be very cumbersome compared to their emergence [16]. Almost all countries that have signed the UN Drug Conventions have implemented specific listing, with some countries applying generic and/or analogue control in addition to specific control [17].

*Generic definitions* are based on a molecular core structure, which in itself does not necessarily need to be controlled, but upon which particular substituent groups are arranged at specific positions, which describe substances to be controlled [16]. In 2009, the United Kingdom was the first country to incorporate generic definitions into its national drug control legislation in response to first-time identifications of NPS, and many other countries have followed since [17, 18]. Generic controls are undoubtedly a powerful tool for efficiently capturing a wide range of substances. However, it can also be too broad and unspecific. Especially in terms of synthetic cannabinoid (or more precisely synthetic cannabinoid receptor agonist) redesign, generic controls have an enormous scope, far greater than for the already relatively large families of phenethylamines or cathinones. Due to the complexity of the chemical structures, there are therefore certain limits to what can be achieved with generic definitions, also taking into account comprehensibility for non-chemists [18].

Finally, *the analogue approach* is applied to control substances based on their similarity to substances already controlled by law, in terms of their chemical properties or their intended psychoactive effect [16]. Compared to the other two approaches presented, the analogue approach is less popular internationally as it is considered less satisfactory from a legal point of view [16, 17].

To explicitly address NPS, several countries have implemented NPS-specific legislation to complement traditional control mechanisms, with Ireland leading the way by introducing the Psychoactive Substances Act in 2010 [17, 19]. In this context, Ireland and other countries—most recently Australia (2015) and the United Kingdom (2016)—have adopted effects-based definitions that refer to the capability of a substance to influence the user’s mind, mood, brain, or behaviour [20]. Kavanagh and Power (2014), for example, stated that changes in Irish NPS legislation specifically targeting suppliers and vendors led them to change the content of their products from now controlled to non-controlled substances [19]. However, Smyth et al. (2017, 2019) found that this legislation was also accompanied by a decline in NPS use disorders and drug-related psychiatric admissions [21, 22]. Stevens et al. (2015) and many others have heavily criticised the British Psychoactive Substances Bill [23]. Thus, Reuter and Pardo (2016) summarised that the problems of a total ban, as proposed by the law in relation to NPS, may be inherent, as the definition of psychoactivity is conceptually problematic. Operationalising psychoactivity as a useful concept for legal control purposes is extremely challenging, perhaps even impossible, and the disconnection of penalties for offences against a total ban from the determination of a substance's harmfulness is normatively questionable [24].

Furthermore, the European Union (EU) has made considerable efforts to establish a transnational framework for addressing the NPS problem. Although the EU has rarely relied on legally binding instruments to align the drug laws of the different member states, the implementation of transnational networks and ‘soft law’ measures (such as guidelines) have contributed to the harmonisation of domestic policies and policy convergence between the EU member states and beyond [25]. However, the national legal responses of the various member states continue to vary to some extent, not least because of the many national peculiarities.

The prevalence of NPS use in the previous 12 months in Germany is estimated at between 0.7 and 1.4%, depending on the federal state, and thus appears to be slightly higher than the European average, with overall low use in the general population compared to other traditional drugs [26–28]. To counteract NPS distribution, newly emerging substances of particular concern were initially only enumeratively included in Annexes I to II of the Federal Narcotics Act (BtMG), thus following a specific listing approach. As the legislators could hardly respond to the large number of new releases of NPS within a short period, the prosecution of some substances, or more precisely synthetic cannabinoids, not explicitly covered by the BtMG often relied on the application of the Medicinal Products Act (AMG). Thus, Germany was in good company, as many European countries initially reacted by applying consumer protection or medicinal laws to remedy the situation [15]. However, with the ruling of the European Court of Justice on 10 July 2014, according to which synthetic cannabinoids are not covered by the term ‘functional medicinal products’ [29] and that this can also be applied to other NPS, this was no longer possible. To close the legal gap created by this decision, the New Psychoactive Substances Act (NpSG) was introduced on 26 November 2016 [30].

This law has several special features that had a certain novelty within the framework of German drug control legislation, such as a generic approach. At the time of its introduction, the NpSG targeted synthetic cannabinoids, phenethylamines, and cathinones, thus covering most of the NPS on the market at that time. Although NPS that prove to be particularly hazardous continue to be included individually in the Annexes to the BtMG, the NpSG now provided an additional legal instrument to cover entire substance groups. Another national legal novelty is the extent to which the law results in sanctions (e.g. imprisonment, fines). In other words, the acquisition and possession of NPS for personal use are prohibited but are generally not prosecuted or penalised. Rather, criminal prosecution and penalties only apply to offences such as trafficking, selling, or manufacturing NPS. Accordingly, the penal provisions are also directed only at these offences and are sanctioned by a term of imprisonment of up to 3 years, up to 10 years in certain aggravating circumstances, or a fine. Regardless of criminal prosecution, however, there is always the possibility of seizing and destroying the substances in question [31]. The prohibitions thus form, in a sense, a hybrid regulation of consumer protection and prohibition law. Concerning criminalisation and the range of penalties, the law is largely based on the AMG, while the terminology and the system are predominantly adopted from the BtMG [30].

Depending on the individual case, the BtMG may impose higher penalties than the NpSG, namely up to 5 years of imprisonment, up to 15 years of imprisonment in certain aggravating circumstances, or a fine [32]. As mentioned above, this is because only particularly dangerous NPS are placed under the BtMG. Therefore, the NpSG—analogous to its counterparts in other countries [20, 33]—does not apply to narcotics as defined in the BtMG (nor to medicinal products as defined in the AMG). Furthermore, the handling of NPS for commercial, industrial, and research purposes is exempted [31].

With the introduction of the NpSG, the German legislators intended to combat the spread of NPS and restrict their availability to protect the health of individuals and the population, especially adolescents and young adults. Given the changed legal situation, the question arose as to what effects this would have on those directly or indirectly affected by the new law, namely NPS users, staff of addiction services, and members of law enforcement authorities. In this context, it seemed interesting to contrast the three perspectives and look for similarities and differences in the perception of the NpSG to better understand potential areas of tension, for example with regard to the use of illicit substances and the degree of repression, and to be able to evaluate different strategies in dealing with NPS more concisely [34].

The first objective of the present study was to examine the extent to which the different groups of people were familiar with the new law, regarding both controlled substances and criminal liability. The second objective was to investigate whether the three groups noticed any changes that they attributed to the introduction of the NpSG, for example in terms of patterns of use or the market situation, and also to be able to assess whether the legislation (in the analogy referred to as the ‘cat’) has succeeded with the NpSG in counteracting the efforts of the manufacturers (i.e. the ‘mouse’). Our third objective was to gather opinions on the new law, particularly concerning its specific features and its practicability, to eventually derive recommendations for German NPS legislation based on the three different perspectives and to contribute to the body of international experience in dealing with NPS, especially by applying generic definitions.

## Methods

Owing to the complexity of the subject and to gain profound insight, we chose a qualitative approach. To assess the effects of the introduction of the NpSG on those affected, semi-structured narrative in-depth interviews were conducted as part of a multicentre study throughout Germany with representatives of three different groups of people: users of NPS, staff of addiction services, and members of law enforcement authorities. The interviews were a central component of a large-scale project funded by the German Federal Ministry of Health to evaluate the impact of the introduction of the NpSG conducted between June 2017 and August 2019. Further details on the methodology can be found elsewhere (Kraus et al. [30]).

### Participants

The 113 NPS users were on average 29.5 years old (*SD* = 8.9; *range* = 18–53), among them 11.5% women and 88.5% men. About half of them (52.2%) were recruited via addiction care facilities, which they had, however, not visited (only) because of an NPS problem. The rest were recruited through other channels (see below), resulting in a sample ranging from marginalised drug users who used drugs on a daily basis to recreational users, including those who primarily want to intensify their party experience by using stimulating NPS and those who are keen to experiment and to explore novel effect profiles (hereafter referred to as ‘psychonauts’). For more details on the characteristics of the NPS users, see the results section.

A total of 33 staff members from addiction care facilities were interviewed, most of them working in addiction counselling, followed by low-threshold and inpatient treatment facilities. The facilities were primarily specialised in illegal substances and, in some cases, offered youth counselling in addition to counselling for parents and relatives. Some of the interviewees were also engaged in prevention projects active in the party scene.

The 47 interviewed members of law enforcement authorities worked for the police, customs, and prosecution. The initial plan was to interview not only law enforcement authorities but also the courts on the practical handling of the NpSG. However, it soon became clear that, in light of the very early evaluation of the impact of the NpSG, corresponding criminal proceedings were not yet pending in court. For this reason, the judges were not expected to have any experience with the NpSG and, hence, were not interviewed.

### Procedure

The participants in the three groups were sampled purposively. For the group of users, a minimum age of 18 years and NPS use within the last 12 months were set as inclusion criteria. To reach a diverse range of NPS users and to ensure a certain heterogeneity of the sample regarding consumption patterns and motives, recruitment was carried out within different settings using several techniques. A large part of the users was recruited through addiction care facilities. Further, users were reached via the Internet; calls for participation were issued, for example in scene-relevant fora, in an article in an online magazine about the changes in the NPS legislation, and in the newsletter of an online NPS shop. In addition, with the help of prevention projects active in the party scene, flyers inviting users to participate in the study were distributed at festivals and other events. These flyers were also placed in cafeterias of universities, in student residences, in late-night, head and grow shops, in medical-psychological examination services to regain the driving licence, in event halls, bars and clubs and as a supplement to a music journal. Apart from that, the snowball principle was applied, i.e. some users participated on the recommendation of those already interviewed.

When recruiting staff of addiction care facilities via different study centres, it was ensured that the interviewees had regular contact with NPS users and had already familiarised themselves with the central contents of the NpSG prior to the interview.

The members of law enforcement authorities were selected based on their professional role and special knowledge in the field of drug-related crime. To be able to make statements about the NpSG, the selection process also took into account the fact that the officials were involved in the prosecution of BtMG and NpSG offences in their everyday work. For this reason, most of the interviewees were contacted directly via the corresponding Ministries of Interior or Justice of the different federal states. Additionally, some members of law enforcement authorities were recruited through referrals from officials named by the ministries.

In advance of the interviews, participants gave informed consent regarding the objectives of the study, audio recording and data protection. Owing to considerable geographical distances, telephone interviews had to be conducted in many cases, especially with the members of law enforcement authorities. However, most of the NPS users recruited via addiction care facilities were interviewed face-to-face, as were the staff of these facilities.

The interviews with NPS users were preceded by a short questionnaire on consumption patterns and demographics. The research staff have been trained in the use of the topic guides tailored to the respective groups of people. Each of these consisted of different thematic complexes to which open questions were subordinated. The interviews focused on the groups’ particular experiences with the NpSG and included questions on perceived changes associated with the introduction of the NpSG as well as questions on opinions regarding legal and criminal policy issues. The interviews with the NPS users lasted on average 30 min and those with the staff of addiction care facilities and members of law enforcement authorities were on average 45 min. All interviews took place between November 2017 and July 2019. NPS users were reimbursed with a 20-euro shopping voucher.

### Data analysis

The interviews were audio-recorded before being transcribed verbatim. The analysis of the pseudonymised transcripts was performed content-based according to Mayring [35] using MAXQDA, special software for qualitative data analysis [36]. A separate code system was created for each group of people. The structure of the three code systems loosely followed that of the corresponding topic guides. The codes were derived inductively from the data and branched out further in the course of the analysis process until data saturation was reached. The code definitions were specified as precisely as possible in memos to ensure that the text segments are classified according to a consistent logic, even across several coders. For reliability purposes, about a quarter of the interviews were coded by two different coders. For some topics, where relevant, interviews with users were analysed according to recruitment setting, and interviews with staff of addiction care facilities and law enforcement authorities according to the regional origin, i.e. north, east, south, and west Germany. The questionnaires were analysed using SPSS 22 [37].

## Results

### Characteristics of the NPS users

**Fig. 1:**
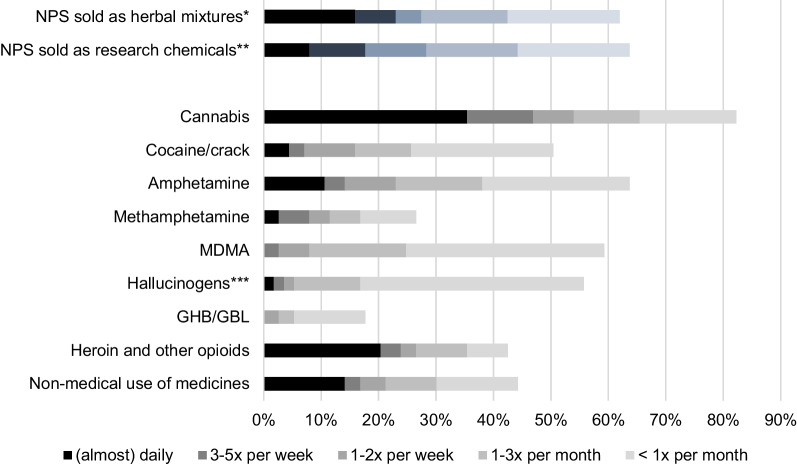
Frequency of use of NPS and other psychoactive substances within the last 12 months. *incl. c-liquids, **incl. ‘bath salts’, ***LSD, DMT, 'magic' mushrooms, ketamine, PCP, etc.

Almost all of the 113 NPS users (96.5%) stated in the questionnaire that they had used other psychoactive substances in addition to an NPS within the last 12 months. Figure 1 describes the frequency of use of each of these substances. Overall, 82.3% of the NPS users reported having used cannabis in the last 12 months and for 35.4%, cannabis was the substance most frequently used (almost) daily.

Figure 1 also illustrates the frequency of NPS use, organised by those sold as herbal mixtures and those sold as research chemicals. About the same number of users reported having used herbal mixtures (61.9%) or research chemicals (63.7%), with the former being used more frequently on an (almost) daily basis (15.9% vs. 8.0%).

Table 1 provides further details on the herbal mixtures and research chemicals used by the participants within the last 12 months and shows that cannabinoids (61.9% + ≤ 8.8%), which were most likely also present in all herbal mixtures, dominate, followed by phenethylamines (37.2%) and cathinones (26.5%).

**Table 1:** Use of herbal mixtures and research chemicals within the last 12 months

	% (Count)
NPS sold as herbal mixtures	
Ready-made mixtures	53.1 (60)
Home-made mixtures	7.1 (8)
C-liquids	8.0 (9)
Other	6.2 (7)
NPS sold as research chemicals	
Cannabinoids	8.8 (10)
Cathinones (incl. 'bath salts')	37.2 (42)
Phenethylamines	26.5 (30)
Opioids	15.0 (17)
Benzodiazepines	13.3 (15)
Arylcyclohexylamines	10.6 (12)
Tryptamines	8.8 (10)
Other	23.9 (27)

The participants used NPS for the first time at an average age of 24.7 years (*SD* = 9.6; *range* = 8–48), with most of them naming herbal mixtures as being their first used NPS product (64.6%).

### Awareness of the introduction of the NpSG

More than half of the NPS users stated in the interviews that they had already familiarised themselves with the NpSG prior to the interviews, albeit to varying degrees. Psychonauts, in particular, had detailed knowledge of the substances subject to the NpSG and were sometimes able to correctly name aspects of criminal liability, but in some cases reported ambiguities or uncertainties regarding practical handling. NPS users recruited via an addiction care context, some of whom showed patterns of high-risk drug use, were much less familiar with the contents of the law and often justified this by the lack of relevance to them.

‘You hear about such laws now and then. But on the street, it just doesn't make any difference.’ (NPS user)

Most NPS users, however, expressed great interest in the NpSG.

The interviews with staff of addiction care facilities from different federal states revealed geographical a north–south and an east–west divide, insofar as the interviewees from the southern and western part of Germany had a much more differentiated knowledge of the relevant legislation. This was also because, in the course of their work, they were confronted with NPS users more frequently than their colleagues from the north and east of Germany. A large number of the contacted staff members from addiction care facilities in the north and east had either not heard of the law and/or had almost no experience in dealing with NPS users and were therefore excluded from participating in the study.

The north–south divide was also reflected in interviews with members of the law enforcement authorities. Accordingly, respondents in the southern part of Germany reported more proceedings and crackdowns. The interviews also indicated that the introduction of the NpSG poses a new challenge for law enforcement, as it is difficult to separate violations of the BtMG and the NpSG at the beginning of an investigation, especially as some substances subject to the NpSG have meanwhile also been included in the Annexes to the BtMG and will continue to be so. To prevent any potential uncertainties in handling, according to all interviewees, when detecting suspicious substances, a potential violation of the BtMG is generally assumed.

‘When I'm holding one of these sachets in my hand, I naturally don't know what's in it from the outside. So we have the instruction to say, “First we report narcotics according to the BtMG, submit [the sachet] to get an expert opinion, i.e. from a chemist who examines it, and then we learn whether the substance is subject to the BtMG or the NpSG.”’ (police officer)

### Perceived changes

In connection with the introduction of the NpSG, the respondents from all three groups noticed considerable evasive movements concerning the NPS products offered on the market, which in some cases also affected user behaviour.

The NPS users frequently mentioned that the introduction of the NpSG was accompanied by a reduction in the quality of the NPS and—depending on the type of products and the source of supply—either a decrease or increase in potency. This was in line with the observations made by staff members from addiction care facilities, although they primarily described an increase in potency. In this context, members of law enforcement authorities found that seized preparations, besides substances controlled by the NpSG, increasingly contained NPS that were already subject to the BtMG. Furthermore, they reported the emergence of completely new substances on the market that were not covered by either the BtMG or the NpSG. Cumyl-PeGaClone, which was also well known to the other two groups, was often cited as an example. Thus, a public prosecutor reported in connection with a major trial:

‘In about 40% of the cases, we had narcotic contents, in 23% of the cases contents [covered by the NpSG but not (yet) by the BtMG], and in 27% of the cases this Cumyl-PeGaClone which was not yet listed in [the Annexes to the BtMG] at the time.’ (prosecutor)

The members of law enforcement authorities mostly referred to detected herbal mixtures and emphasised that the online shop involved promoted the legality of the respective products, although they contained narcotics. Concerning synthetic cathinones, they stated that they had identified significantly fewer.

Most NPS users reported a reduced availability of established NPS and/or an impairment of their supply channels as a result of the relocation of the headquarters of German online shops abroad. Although the latter could not be confirmed by members of law enforcement authorities, they noticed, however, that since the NpSG came into force, the locations of online shops have mostly been obscured by pseudo addresses (also abroad) in the imprint. Nevertheless, the websites continue to be designed as supposedly German sites and assure that the goods are shipped from Germany.

‘We have not noticed that the internet shops are moving abroad. They rather think they're safe. In any case, it is actively promoted that they are always up to date and that the user is not at risk of criminal prosecution.’ (police officer)

According to NPS users, these developments have led many to switch to novel or unknown NPS, which are also perceived as more hazardous, to return to the controlled original and/or to change supply channels, although this may involve more health and criminal risks.

‘With the new law, I think the more harmless substances have completely disappeared from the scene; now there's only such hardcore, highly bred stuff. It's sold freely, which I don't approve of at all. With a substance—an opioid, bromadol—when I read that you should wear a full-body protective suit and a respiratory mask because if you get a crumb of it on your skin or breathe it in, you die because it is so highly potent, that's where the fun stops for me. And that's just now after the law was brought out, that has worsened, I think. Because now there are so many substances that are so highly potent, and there are probably so many people who have no idea about them and think, “Yeah, that's like something illegal, the classic stuff that you know.”, and then they overdose and die.’ (NPS user)

However, members of all three groups reported that overall NPS consumption tended to decline (with some respondents excluding the southern part of Germany). Yet, only in very few cases did they attribute this directly to the introduction of the NpSG, but rather to a changing image of NPS, for example.

### Opinions and expectations

About the assessment of the NpSG, the three groups agreed on a variety of points, although with differently weighted nuances. The majority of mainly NPS users regarded the described evasive movements as resulting from the more complex market situation, caused by the introduction of the NpSG and leading to riskier consumption patterns, as highly critical. Therefore, they came to an overall negative conclusion.

‘It [the NpSG] tries to regulate a market that cannot be regulated—not even with bans. So, it just creates new problems; like with a Hydra—you just cut off one head, but then four new ones grow.’ (NPS user)

Referring to the content of the law, interviewees from all three groups noticed discrepancies between theory and practice. In their experience, not much has changed for NPS users. At least initially, one is still ‘treated like a criminal’ (quote from an NPS user), as substances are usually seized and destroyed preventively, on account of uncertainty as to the nature of the substance (BtMG/NpSG) involved in the situation of detection and to avert an imminent danger. Accordingly, there is always an initial suspicion of a criminal offence under the BtMG, and a corresponding procedure is initiated.

Members of law enforcement authorities and some staff members from addiction care facilities commented positively on the precise and efficient control of substances under the law. However, participants from all three groups criticised how the classification of NPS according to the laws to which they are subject is not very straightforward.

Although the other two groups rather regretted this fact against the background of the generally prohibitive course of German drug policy, members of law enforcement authorities appreciated the existence of an additional legal instrument, precisely for the elimination of legal uncertainties regarding the handling of NPS and for the possibility of preventive seizures. However, in this context, NPS users sometimes indicated that the distinction between personal consumption and trafficking may not always seem to be clear, although most of them even considered it appropriate to prosecute NPS trafficking.

‘I don't get the feeling that clear procedures are being followed.’ (NPS user)

Even though some misunderstandings with NPS users became apparent in this context, the majority of all interviewees eventually welcomed the idea of not criminalising the acquisition and possession of NPS. However, some of them also argued that no prosecution at all would send the wrong signal, given that in some cases, the substances are seriously harmful to one’s health.

In terms of the criminal prohibition, or more precisely the level of penalties, interviewees from all three groups commented on the disproportionality between NpSG and BtMG, considering the assumed risk potential of the substances subject to the respective laws.

‘Well, to be honest, I'd do it the other way round. Because cannabis is a lot safer than this stuff!’ (NPS user)

The staff of addiction care facilities often emphasised that resources tied to law enforcement were generally better invested in prevention work. Some police officers were also in favour of strengthening prevention work in this context but left it at that

Overall, the vast majority of respondents did not expect the introduction of the NpSG to bring any significant changes, but rather that the ‘cat-and-mouse game’ would continue.

‘They [the supplier] […] are always prepared for upcoming bans and already have something up their sleeve. Even now, when [Cumyl-PeGaClone] was placed under [control], it had been expected six months earlier and a follow-up substance was ready for use. And, by the way, the follow-up substance for this one is also already in the pipeline. So much for this cat-and-mouse game.’ (NPS user)

‘It's the same cat-and-mouse game as always, except that the response is faster and certain substance groups are included. But perhaps it is simply too early to make any statements about this.’ (police officer)

## Discussion

The introduction of the NpSG was accompanied by several changes, which have been examined in the present study from different angles—by NPS users, staff of addiction care facilities, and members of law enforcement authorities—to provide an overall picture that allows further considerations on how to deal with NPS in Germany and beyond.

Among NPS users, most had used at least one other psychoactive substance within the last 12 months, with cannabis being reported most frequently. A relatively high percentage also reported the use of synthetic cannabinoids, mostly in form of herbal mixtures. This pattern matches trends observed in Europe, where synthetic cannabinoids in form of herbal plant material represented the majority of NPS on the market at the time of data collection [30].

NPS users and the staff of addiction care facilities in certain regions were generally familiar with the NpSG only to a limited extent. In the interviews with NPS users, it became clear that, although they are in principle interested in the law, many have resigned themselves to not understanding it because of its complexity. However, this may also be attributed to the lack of relevance of the law in their everyday life, especially on the drug scene. Concerning controlled substances, staff of addiction care facilities and members of law enforcement authorities also had to admit that the classification of substances under the current law is not particularly easy to understand. This coincides with the opinions of various critics who argue that a generic approach may lead to uncertainties about which substances are controlled, not only for those who risk criminal sanctions but also for those who enforce legislation [1]. Except for some psychonauts, aspects of criminal liability could rarely be described by NPS users. This may also be because, due to the effects of changes in NPS legislation on the market, the selection of controlled substances is much more tangible for the users than aspects of criminal liability. These may appear somewhat more abstract, especially since court rulings tend to be reserved for a particularly interested audience.

Unfortunately, the present study could not conclusively clarify the reason for the regional imbalance among the staff of addiction care facilities regarding their knowledge of NPS legislation and the frequency of their interactions with NPS users (in the sense that NPS play a greater role in the south and west of Germany than in the north and east). It could be that NPS are less prevalent in the north and east, at least among the addiction care clientele, although Gomes de Matos et al. (2018) found no evidence for this in an epidemiological study [26]. In contrast, data on the use of addiction care and NPS seizures indicate a north–south and an east–west divide [30].

In any case, it seems reasonable to intensify educational measures for NPS users and ‘those interested in use’ (within the framework of selective and indicated prevention) concerning NPS and the associated legislation, preferably in combination with a wide range of harm reduction methods. Ideally, this would be accompanied by raising the awareness of professionals nationwide, especially staff of addiction care facilities, and the establishment of targeted group-specific training measures.

In all three groups, the majority of respondents did not consider the introduction of the NpSG to be particularly effective in terms of the market situation. Some interviewees perceived a decrease in quality and—depending on the type and origin of the product—a decrease or increase in potency. This may be related to the fact that since the introduction of the NpSG, completely new substances emerged and more preparations containing NPS that were subject to the BtMG were seized. Concerning synthetic cannabinoids, these observations are consistent with the findings from a systematic market monitoring, where test purchases from 2015 to 2018 were analysed [30]. Further evidence is provided by forensic analyses of NPS seizures that indicate novel substances appeared primarily as cannabinoids, whereas new structural variations in phenethylamine or cathinone derivatives were not observed in the period under consideration until the beginning of 2019 [30]. At least seven of these novel cannabinoids were also first-time identifications at the EU level [38].

After the introduction of the NpSG, there was an increase in preparations in circulation that contained NPS that were subject to the BtMG. This change in ingredients could be because it was no longer a major difference to manufacturers and suppliers under which law they are prosecuted. It can be assumed that the criminal prohibition of NPS trafficking, at least indirectly through its strong influence on the market, undermines the idea of non-criminalisation inherent to the NpSG (i.e. that no penalties are imposed for the simple acquisition and possession of NPS for personal use).

In the interviews, some of the novel substances that intentionally circumvent the NpSG and were presumably designed for the German market were specifically mentioned including Cumyl-PeGaClone and 5F-Cumyl-PeGaClone. Owing to their particularly hazardous nature, both substances were placed under the BtMG in July 2018 and July 2019, respectively [39]. Since an extension of the cannabinoid group under an ordinance amending the Annex to the NpSG and Annexes to the BtMG, such substances are also covered by the NpSG. The ordinance came into force in July 2019 and, in August 2019, a herbal mixture containing Cumyl-CBMICA was seized [39]. Until then, the side chain defined in the NpSG on synthetic cannabinoids covered five to seven ring atoms, which means that Cumyl-CBMICA, containing only four atoms, was not included [40]. Another amendment concerning the NpSG in July 2020 closed this gap and also anticipated conceivable variants with three rings [41]. In July 2020, a test purchase was made of a sample containing Cumyl-BC-HpMeGaClone-221, which shares structural similarities with Cumyl-PeGaClone. Cumyl-BC-HpMeGaClone-221, however, was not covered by the legislation in force at the time due to a carbon ring attached via a methylene bridge [38, 42]. This gap has been responded to with the latest amendment which came into force in July 2021, resulting in further completely new substances appearing on the market shortly afterwards [38, 42].

This illustrates that the cat-and-mouse game continues, but it gets more and more complex as the players involved take bigger steps at each point. Furthermore, the playing field is becoming increasingly smaller. Besides the extensions of the law concerning the cannabinoid group, there were also extensions concerning the phenethylamine group. Also, five new groups of substances were added to the law: benzodiazepines, tryptamines (e.g. LSD derivatives), N-(2-aminocyclohexyl)amides (synthetic opioids such as U-49900), arylcyclohexylamines, and benzimidazoles. However, these additions to the NpSG were also accompanied by the appearance of some substance novelties, e.g. LSD derivatives (‘legal LSD’) such as 1V-LSD. Nevertheless, data on seizures strongly suggest that the number of NPS on the market that are not subject to any legislation is decreasing [43]. In return, offences committed in connection with NPS are increasing, both use-related offences (BtMG: 2609 in 2018 and 2925 in 2021) and trafficking offences (BtMG: 293 in 2018 and 726 in 2021; NpSG: 495 in 2017 and 771 in 2021) [44].

Ultimately, the outcome of the game remains uncertain, especially since the German market is not only driven by German legislation. Accordingly, Halter et al. (2020) noted corresponding changes in the prevalence of 5F-ADB and structurally closely related synthetic cannabinoids in connection with Chinese legislation [45].

Interviewees from all three groups reported a decline in NPS use (possibly except in southern Germany). This may be less related to the introduction of the NpSG than to all the changes made in NPS legislation taken together, including the amendments to the BtMG to incorporate individual NPS. The effects on the quality and variety of substances on the market may have contributed to an increasingly poor image of NPS, which has also been fuelled by the media.

However, the described decline in NPS use is not reflected in population data. This is evident in data from adults (18 to 64 years) where 12-month prevalence rates of NPS use of 0.9% were found for both 2015 and 2018, as well as in data from adolescents/young adults (12 to 25 years) with 12-month prevalence rates of 0.0% for 2015 and 0.1–0.2% for 2019, respectively [46–49].

Hence, with the introduction of the NpSG, the legislators may have succeeded to some extent in combating the spread of NPS. However, this has also resulted in novel, completely unknown substances on the market, for which users are even less able to assess potential harms than for the known ones. According to the interviewees, this has led some users to return to established substances, which on the one hand may be accompanied by criminal prosecution, but on the other hand, may also make it easier to assess the health risks of drug use. It has also resulted in some users switching to other supply channels, opening themselves up to unknown risks.

As far as the NPS market is concerned, the changes in legislation have been accompanied by both positive (‘intended’) as well as negative (‘unintended’) impacts. Concerning the latter, especially the conglomeration of these novel substances with their unpredictable health risks and side effects, it is essential that the relevant actors remain alert. This also underlines the importance of international cooperation, be it in scientific research (i.e. collecting analytical, pharmacological, and toxicological data on substances newly appearing on the international market), in improving early warning systems, or in the control of substances to better cope with the dynamics of the NPS market [50].

While the interviewed NPS users and the staff of addiction care facilities generally tended to deplore the introduction of the new law in light of the rather prohibitive spirit of German drug policy, the members of law enforcement authorities appreciated the NpSG as an additional legal instrument that, in their views, eliminates legal uncertainties and allows for the preventive seizure of substances.

Across all three groups, the interviews revealed the impression that, despite the welcome intention of the law to not prosecute the acquisition and possession of NPS for personal consumption, not much has changed in practice for NPS users. This relates to the principle of legality in criminal proceedings, which states that law enforcement authorities are obliged to prosecute any suspect and to intervene if there are sufficient indications. For instance, due to the difficulties associated with the classification of seized substances under the current law, a criminal offence according to the BtMG must be assumed until proven otherwise by toxicological findings. Moreover, the distinction between personal use and trafficking does not always seem obvious, which of course will have different consequences for the accused. Therefore, and also to exclude a false signal effect of the NpSG in comparison with the BtMG, as well as to avoid evasive movements, the criminal liability of possession for personal use should be harmonised in both laws. This could be achieved through either general criminalisation or non-criminalisation. The interviews with users and staff of addiction care facilities indicate that both groups prefer the latter, though the majority of all interviewees considered it appropriate to prosecute NPS trafficking.

Against this background and the current debate on cannabis legalisation in Germany, the question arises whether the decriminalisation of cannabis users would also lead to a reduction in the prevalence of synthetic cannabinoids [34]. A recent study investigating the association between synthetic cannabinoid exposures reported to US poison control centres and the status of state cannabis legalisation reveals that the adoption of permissive cannabis law was associated with significant reductions in reported synthetic cannabinoid exposures [51]. However, in order to assess the impact of the possible legalisation of cannabis in Germany, further research will be needed in due course.

Within the framework of the project to evaluate the impact of the introduction of the NpSG, all data sources available at the time were used, with the interviews presented providing a significant contribution. However, there is one major limitation. As the findings have also shown, the interviews were, at least in part, scheduled too early. It takes some time for a law to reach people and for them to have gained enough experience/precedents to be able to make conclusive statements. The presented findings are only a snapshot in time indicating that a repetition of interviews at a later stage is recommended. Moreover, the results of the study are not representative. Rather, the results merely reflect the subjective perceptions and opinions of the participants, which are supposed to illustrate the views of the respective group.

Potter and Chatwin (2018) note that such NPS policies—the introduction of the NpSG being no exception considering its impact—basically represent a shift away from harm reduction and evidence-based policies back towards more stringent measures [52]. Stevens and Measham (2014) describe this as part of the 'drug policy ratchet’: a rapid—or efficient—response is becoming increasingly important, with policy-makers, in the absence of scientific evidence, tending to err more and more on the side of caution [53]. This illustrates that however NPS legislation is constructed, the concept of harm reduction must not be neglected. Given the high number of substances adulterated with NPS in circulation, drug-checking services are of particular importance in this context [12]. Drug checking not only provides the opportunity to immediately warn potential users of particularly harmful substances and to empower them to make safer consumption decisions but also allows for the provision of public health alerts. Thus, drug checking serves as a useful monitoring tool to respond to the highly dynamic nature of the NPS market and to implement other evidence-based health care interventions [54]. Accordingly, in addition to a profound realignment of German drug policy in the sense of decriminalisation, it would be highly recommended to consider the legalisation of drug checking in Germany.

## Conclusions

This study has shown that, concerning the introduction of the NpSG and the content of the law, there are some knowledge gaps in the group of users and, depending on the region, also in the group of staff of addiction care facilities, which should be countered with specific educational work and sensitisation to the topic. Even though the introduction of the NpSG has now taken the cat-and-mouse game to the next level and has significantly reduced the national playing field over the years, including through some legislative amendments, the NpSG has also been accompanied by some unintended effects that should also be increasingly addressed in the international arena. At the same time, it seems advisable to employ a broad spectrum of harm reduction methods, such as establishing legal drug-checking services, and to adopt a decriminalising stance, not only with regard to NPS.

## Data Availability

The datasets generated and/or analysed during the current study are not publicly available due to the protection of the participants' privacy but are available from the corresponding author on reasonable request.

## Abbreviations

AMG (Arzneimittelgesetz): Medicinal Products Act
BtMG (Betäubungsmittelgesetz): Narcotics Act
NPS: New/novel psychoactive substance(s)
NpSG (Neue-psychoaktive-Stoffe-Gesetz): New Psychoactive Substances Act
